# Slow motions in A·T rich DNA sequence

**DOI:** 10.1038/s41598-020-75645-x

**Published:** 2020-11-04

**Authors:** A. Ben Imeddourene, L. Zargarian, M. Buckle, B. Hartmann, O. Mauffret

**Affiliations:** LBPA, ENS de Paris-Saclay, UMR 8113 CNRS, Institut D’Alembert, Université Paris-Saclay, 4, avenue des Sciences, 91190 Gif-sur-Yvette, France

**Keywords:** Biophysics, Structural biology

## Abstract

In free B-DNA, slow (microsecond-to-millisecond) motions that involve equilibrium between Watson–Crick (WC) and Hoogsteen (HG) base-pairing expand the DNA dynamic repertoire that could mediate DNA–protein assemblies. R_1ρ_ relaxation dispersion NMR methods are powerful tools to capture such slow conformational exchanges in solution using ^13^C/^15^ N labelled DNA. Here, these approaches were applied to a dodecamer containing a TTAAA element that was assumed to facilitate nucleosome formation. NMR data and inferred exchange parameters assign HG base pairs as the minor, transient conformers specifically observed in three successive A·T base pairs forming the TAA·TTA segment. The abundance of these HG A·T base pairs can be up to 1.2% which is high compared to what has previously been observed. Data analyses support a scenario in which the three adenines undergo non-simultaneous motions despite their spatial proximity, thus optimising the probability of having one HG base pair in the TAA·TTA segment. Finally, revisiting previous NMR data on H2 resonance linewidths on the basis of our results promotes the idea of there being a special propensity of A·T base pairs in TAA·TTA tracts to adopt HG pairing. In summary, this study provides an example of a DNA functional element submitted to slow conformational exchange. More generally, it strengthens the importance of the role of the DNA sequence in modulating its dynamics, over a nano- to milli-second time scale.

## Introduction

DNA–protein recognition processes occur through so-called direct and indirect readout of DNA by proteins. The formation of nucleoprotein complexes requires in particular, recognition of DNA chemical patterns specific to each base, and DNA structural and dynamic features that are sequence dependent. Deciphering the dynamics of DNA is not experimentally easy. Nuclear Magnetic Resonance (NMR) has long been, and remains a powerful technique for capturing picosecond dynamics at atomic resolution; more recent developments of relaxation dispersion experiments extend the timescale up to milliseconds and quantitatively investigate slow conformational exchange processes^1–4^.

Initially, the existence of slow dynamic movements in DNA emerged from early NMR data collected on adenines of TpA steps, by detecting an excess linewidth of adenine H2-resonance protons in biologically active DNA sequences^5,6^. Further investigations showed that this linewidth broadening was sensitive to the TpA tetranucleotide sequence context^6–8^. The observation of these resonance experiments was interpreted as being due to a slow exchange between two conformational states^7,9,10^, which could arise because of poor TpA stacking^9,10^.

R_1ρ_ relaxation dispersion NMR experiments have led to major advances in this field. These experiments were used to reveal and characterize slow conformational equilibria between major and minor conformational states, assimilated to ground and excited states in analogy with spectroscopy. The use of double ^13^C and ^15^N labelled molecules allowed the detection and analysis of excited conformer populations of less than 1%. This approach initially provided insights on various topics essentially related to proteins such as folding, enzymatic catalysis, ligand binding and recognition^1,2,11^, but now also gives information about intrinsic slow motions of nucleic acids^3,12–16^. Applied to B-DNA double helices, this methodology revealed an unexpected equilibrium between two schemes of base-pairing: standard Watson–Crick (WC) base pairs being able to transiently adopt the Hoogsteen (HG) configuration^15,17–20^. Prior to these NMR studies, the first experimental evidence of HG pairing in DNA was obtained in 2002 using a DNA containing 6 A·T base pairs that all crystallized in the HG mode^21^. Both experimental^22^ and theoretical^23^ approaches showed that d(AT)_n_ sequences were globally more stable in the classical WC B-DNA than in a fully HG double helix, at least in dilute aqueous solutions and in the absence of cofactors. However, earlier models^24^ as well as more sophisticated MD structures^15,25,26^ demonstrated that HG pairing can coexist with WC base pairs in the same DNA without generating prohibitive energy cost. Thus, the presence of transient HG base pairs in solution, as shown by NMR, is strongly supported by these previous reports.

Relaxation dispersion NMR studies provided quantitative information that covered three different aspects of the WC ↔ HG exchange process, thermodynamic (conformer populations), kinetic (exchange rates) and structural (chemical shifts of the minor conformer). Modulation of this equilibrium by the base pair type (A·T *versus* G·C) and the sequence surrounding the base pair^15,17,27^ was established, in agreement with, and as a complement to, analyses of H2-linewidth broadening.

That HG or HG-like T·A in TpA steps are found relatively frequently in X-ray structures of DNA duplexes suggests a biological role for the WC ↔ HG equilibrium^27^. In addition to their possible involvement through base-pairing, TpA steps also play a role in nucleoprotein complexes as illustrated by the case of nucleosomes whose positioning along eukaryotic genomes is biased by the DNA sequence. Thus, favourable sequences for forming nucleosomes in vivo as well as in vitro are composed in such a way that A·T-rich and G·C-rich minor grooves generally tend to face towards and away from the histone core, respectively^28–32^. This is particularly the case for the 601 sequence, also called the “Widom sequence”, which is widely used for positioning nucleosomes because of its high affinity for the histone octamer^33^, further enhanced by an enrichment of additional strategically located TpA steps^34–37^. To better understand the properties of the 601 sequence we carried out classic NMR experiments^37^ and modelling^38^ studying four dodecamers that together cover 39 base pairs of the 5′ half of the 601 sequence. One of these dodecamers contains the TTAAA fragment that, in its ground state, shows a remarkable narrowing of its minor groove^37^. This structural characteristic is thought to be associated with an enhanced electronegative potential^39^ that is especially attractive for the histone arginines anchoring the DNA. Given the above, we decided to further extend the exploration of the properties of the dodecamer whose sequence and numbering is given below:

5′- C_1_ C_2_ G_3_ C_4_ **T**_**5**_** T**_**6**_** A**_**7**_** A**_**8**_** A**_**9**_ C_10_G_11_C_12_ -3′

3′- G_24_G_23_C_22_G_21_**A**_**20**_**A**_**19**_**T**_**18**_**T**_**17**_**T**_**16**_G_15_C_14_G_13_ -5′

Here, we will describe NMR experiments, in particular R_1ρ_ relaxation dispersion experiments, that were used to study this dodecamer. Careful analysis and interpretation of the NMR data led to the detection and characterization of slow motions on a patch of 3 successive A·T base pairs in TpA·TpA and ApA·TpT contexts, which participate in WC ↔ HG equilibria. These findings were put into perspective with regard to previous studies in order to discuss the sequence effect on slow motion.

## Results

### 1D spectra of H2 protons

**Figure 1:**
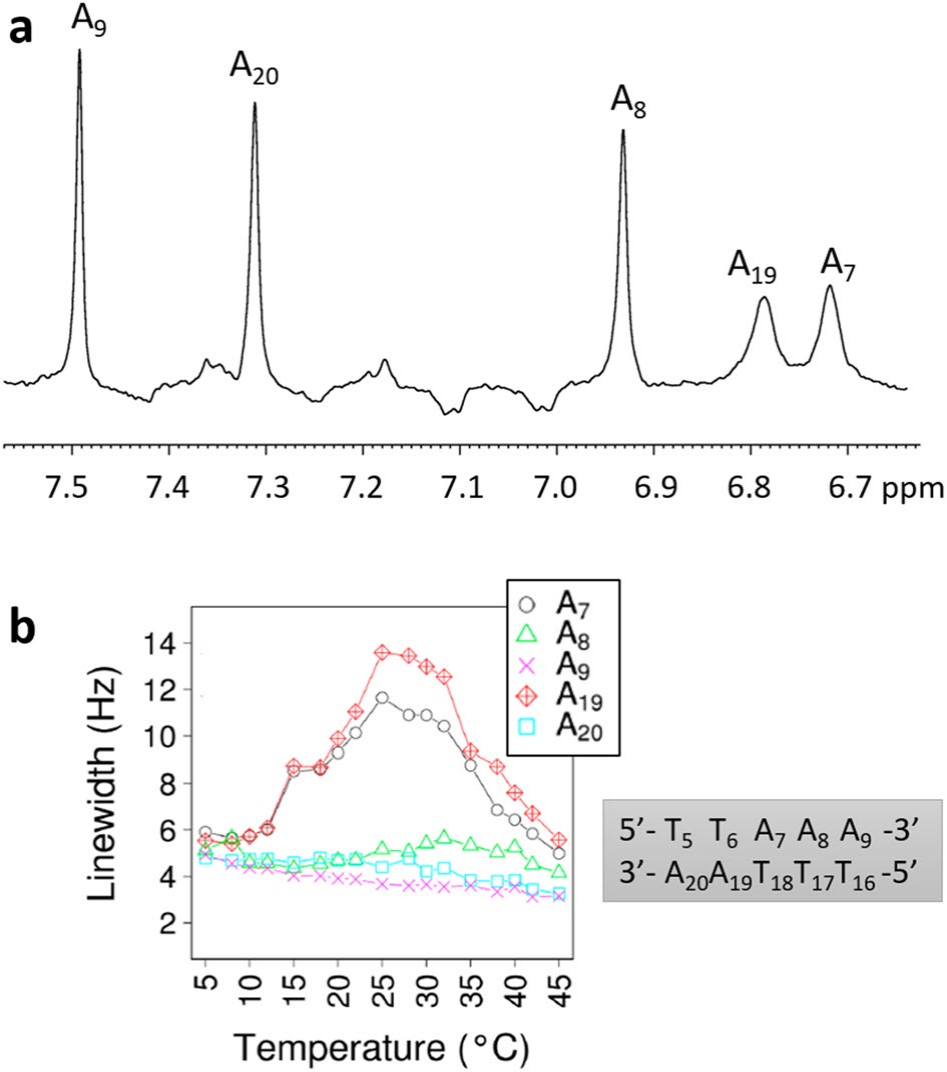
Chemical shifts and linewidth of H2 adenine proton resonances. (**a**) 1D-T1 inversion recovery spectrum of the aromatic region of the unlabelled oligomer at 25 °C in ^2^H_2_O, after having selected the inversion recovery delay to only obtain the H2 resonances of adenines. (**b**) Linewidths (measured at half-width) of H2 adenine proton resonances as a function of temperature. The numbering of the DNA segment of interest is given on the right of the Figure.

In 1D (Supplementary Fig. S1) and 1D-T1 inversion-recovery (Fig. 1a) spectra, the five H2 protons of A_7_, A_8_, A_9_, A_19_ and A_20_ show a very large spectral dispersion compared to those of other non-exchangeable protons. Such dispersion is expected because H2 chemical shifts are extremely sensitive to their sequence environment^6–8^, notably with high-field shifts specific to TpA adenines^9,10^.

Here, the H2 chemical shifts of A_7_ and A_19_ are in fact strongly high-field shifted (Fig. 1a) in the unique TpA_7_·TpA_19_ complementary dinucleotide of the studied dodecamer (see the sequence in above “Introduction” section). Although these adenines share the same tetranucleotide context (TTA_7_A·TTA_19_A), their H2 chemical shifts are clearly different (Fig. 1a). Thus, the sensitivity of H2 chemical shifts to the sequence depends on both 5′ and 3′ nearest neighbours and not only on the 3′ base as previously postulated^10,40^.

A key point is that the H2 resonances of A_7_ and A_19_ are severely broadened compared to those of A_8_, A_9_ and A_20_ (Fig. 1a). These A_7_ and A_19_ H2 broadenings are detected below the Tm of 57 °C (Supplementary Fig. S2) and their amplitudes are maximal between 25 and 30 °C (Fig. 1b). Such behaviour of H2 adenine resonances resembles other TpA results that were interpreted as being the signature for conformational motion^5–9^ occurring at the microsecond-millisecond range^41^.

C2–H2 cross-peaks were then identified from a constant-time ^1^H–^13^C spectrum. Weak intensity and significant broadenings are observed for only A_19_ and A_7_ cross-peaks. 1D H2 inversion-recovery and 1D dispersion R1ρ relaxation spectra show that both H2 and C2 resonances of A_19_ and A_7_ are involved in these specific cross-peak particularities. So, the motions detected here influence the NMR behaviour of at least these two adenine atoms.

**Figure 2:**
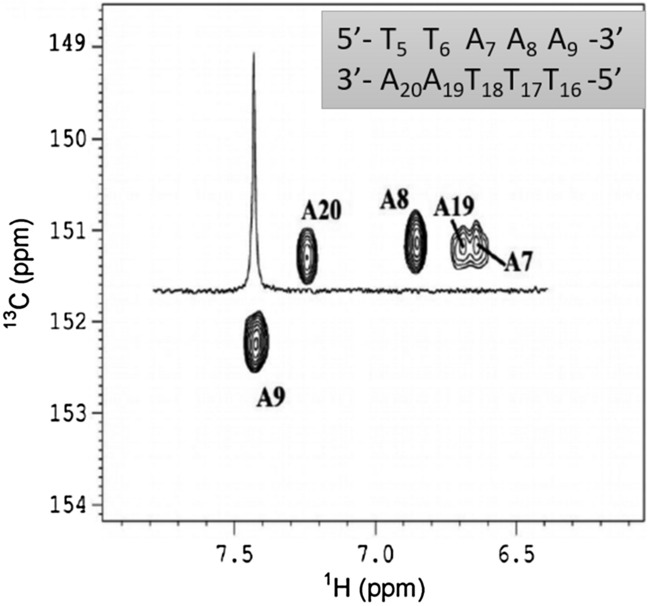
H2–C2 region of adenines of a constant-time HSQC spectrum. The H2–C2 region of adenines of a ^13^C–^1^H constant-time HSQC spectrum (600 MHz) was obtained on the labelled oligomer at 25 °C. The ^1^H 1D spectrum was superposed on the proton frequencies from 1D ^13^C R_1ρ_ dispersion experiment at the ^1^H and ^13^C frequency of A_9_ signal.

The next step was to apply R_1ρ_ relaxation dispersion experiments to the ^13^C/^15^N labelled dodecamer, as a means to follow and describe slow conformational exchanges. The 1D selective R_1ρ_ spectrum of the A_9_ resonance illustrates the good selectivity of this type of experiment, ensuring that the excitation of a given carbon does not affect its spectrum neighbours (Fig. 2).

### On-resonance ^13^C R_1ρ_ dispersion relaxation experiments: evidence of slow conformational exchanges

R_1ρ_ relaxation dispersion experiments^14^ in the on-resonance version constituted a first approach for identifying those carbon atoms of nucleotides submitted to slow conformational exchange. The R_1ρ_ rates were carefully measured to obtain the best estimate of the exponential decreasing ^13^C magnetization. Typical plots of mono-exponential decays of the type shown in Supplementary Fig. S3 illustrate the quality of the data. The R_1ρ_ profiles measured as a function of effective spin-lock field power were fitted using a two-state model and two variables, the relaxation rate R_2_ and the exchange rate R_ex_ (see “Materials and Methods”); 500 runs were performed per fit, to obtain R_2_ and R_ex_ standard deviations.

#### C2 atoms of adenines

**Figure 3:**
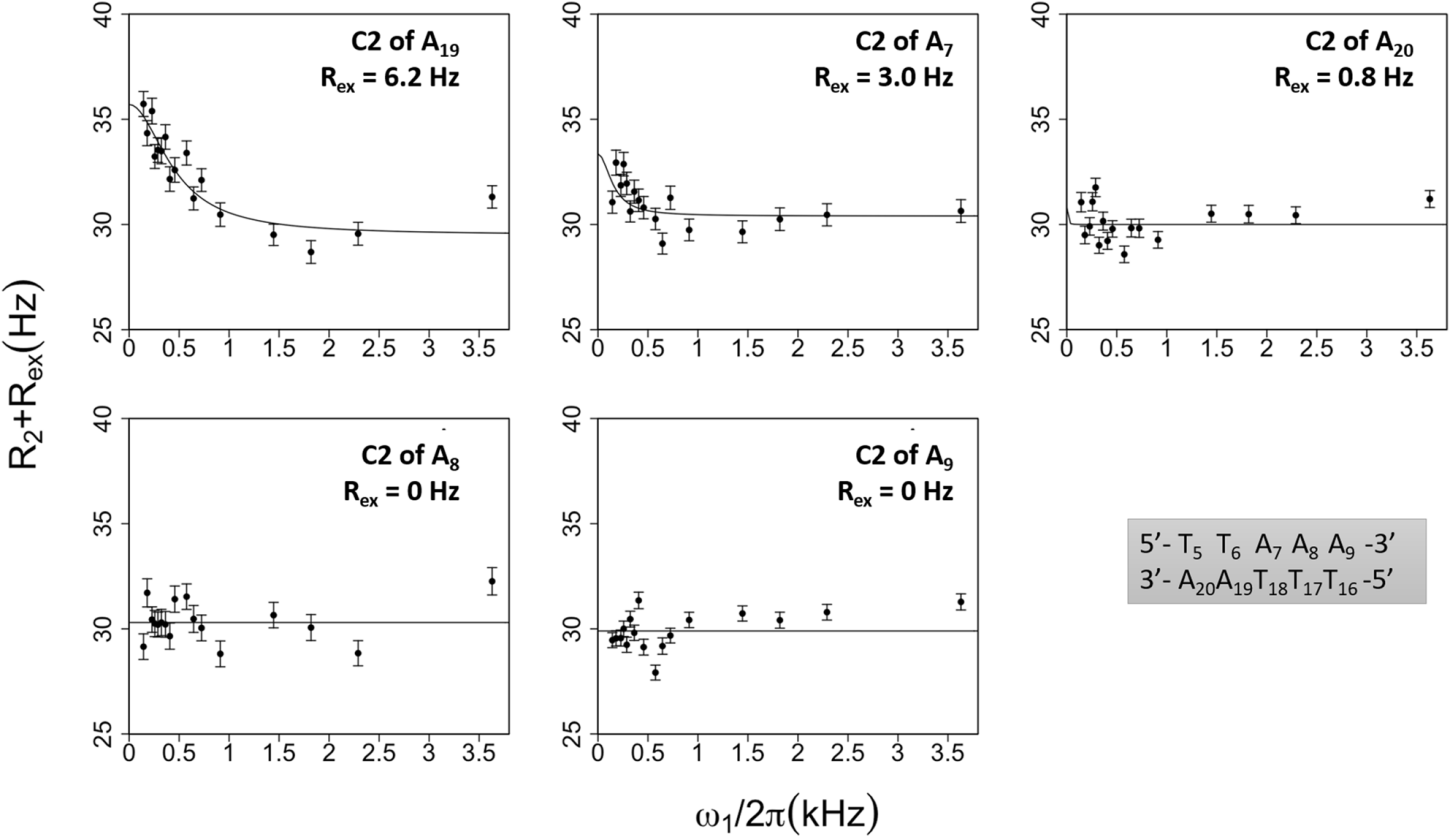
On-resonance R_1ρ_ relaxation dispersion profiles of C2 atoms of adenines. R_1ρ_ (= R_2_ + R_ex_) rates of the C2 atoms of the five adenines were plotted as a function of the effective spin-lock field power (ω1/2π). The experiments were performed at 25 °C. The two-state model fits (solid lines) were obtained using the protocol described in “Materials and Methods”; (R_2_ + R_ex_) standard deviations were calculated from the 500 runs carried out for each fit. The averaged R_ex_ values are specified in each panel. Top: R_ex_ > 0; bottom: R_ex_ ~ 0.

The C2 atoms of A_7_, A_8_, A_9_, A_19_ and A_20_ (Fig. 3) were studied first. Evidence of a slow conformational exchange on A_7_ and A_19_ emerges from the profiles of their R_1ρ_ (= R_2_ + R_ex_) rates measured as a function of effective spin-lock field power (Fig. 3). These profiles and their fits show that C2-R_ex_ is higher for A_19_ than for A_7_ (Fig. 3 and Supplementary Table S2-1 for both R_2_ and R_ex_ values). The consistency between these results and those obtained on H2 adenine atoms (Figs. 1 and 2) argues in favour of a major effect of slow motion on H2 and C2 linewidths. The on-resonance data collected on A_8_, A_9_ and A_20_ C2 atoms reveal invariant R_1ρ_ values (Fig. 3) and thus null R_ex_ values (Supplementary Table S2-1).

#### C6 or C8 atoms

**Figure 4:**
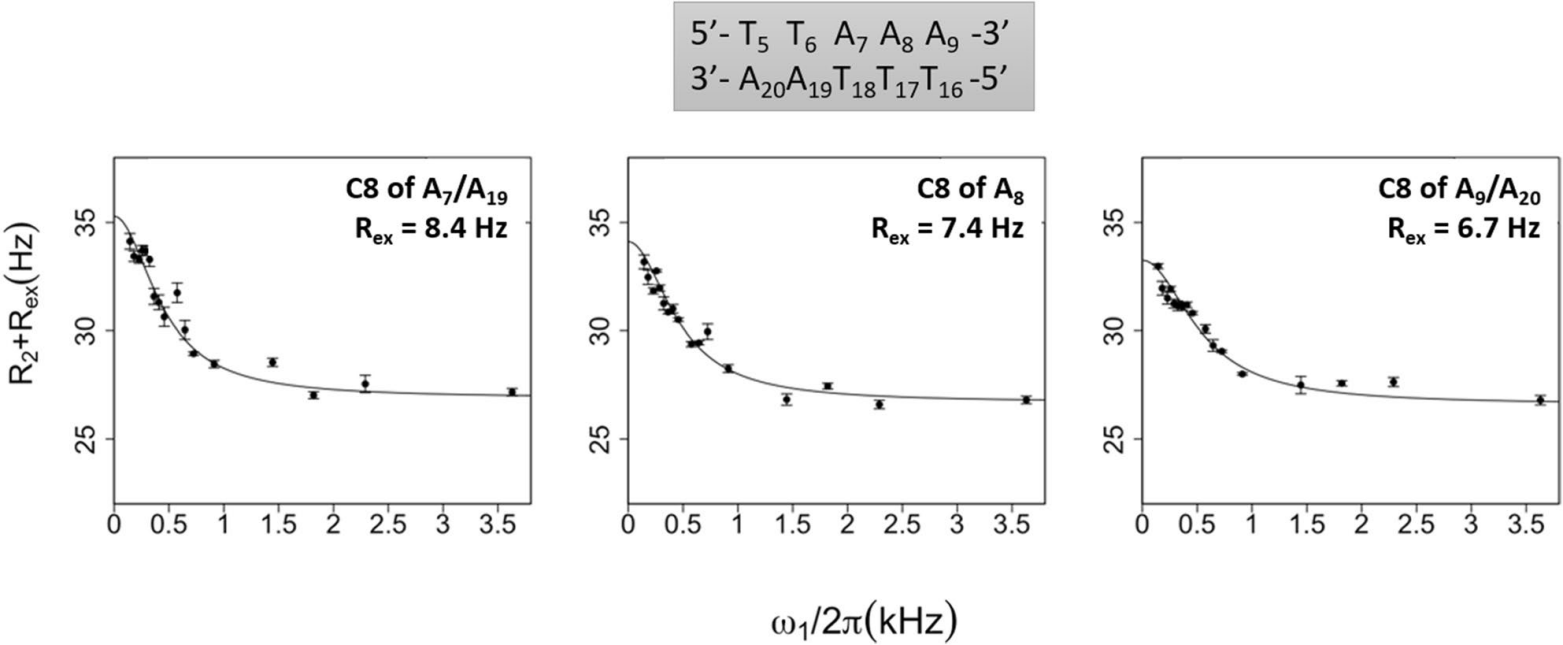
On-resonance R_1ρ_ relaxation dispersion profiles of adenine C8 atoms. R_1ρ_ (= R_2_ + R_ex_) rates of the C8 atoms of adenines were plotted as a function of the effective spin-lock field power (ω1/2π). The experiments were performed at 25 °C. The two-state model fits (solid lines) were obtained using the protocol described in “Materials and Methods”. (R_2_ + R_ex_) standard deviations were calculated from the 500 runs carried out for each fit. The averaged R_ex_ values are specified in each panel.

The C6/C8 on-resonance NMR signals were resolved for over half of the dodecamer nucleotides, comprising A_9_, but severe spectral overlaps occurred for several residues, preventing in particular the distinction of A_7_ from A_19_, and A_9_ from A_20_. The fitting R_2_ and R_ex_ values for the profiles of R_1ρ_ rates as a function of effective spin-lock field power are given in Supplementary Table S2-2. Thus, the existence of a conformational exchange is attested for A_7_ and/or A_19_, A_9_ and/or A_20_, and A_8_ (Fig. 4), with R_ex_ values similar to the highest ones obtained on previous DNA slow motion studies performed in appropriate pH and temperature conditions^15,17,18^. Such events are excluded or at least much more questionable for the remaining nucleotides that display flat R_1ρ_ profiles (examples in Supplementary Fig. S4) and therefore null or low R_ex_ values (< 2.3 Hz) (Supplementary Table S2-2)*.*

#### C1′ atoms

**Figure 5:**
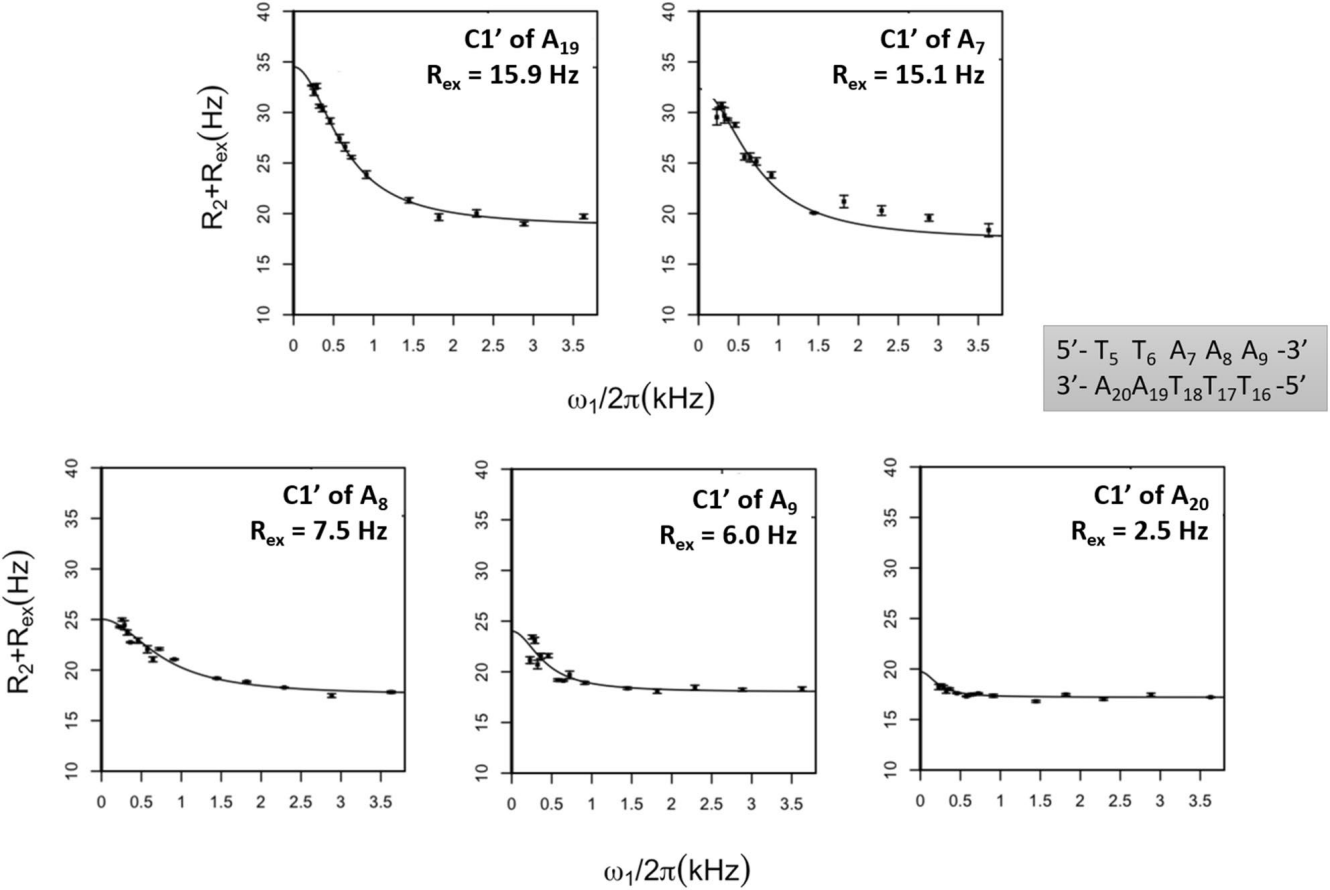
On-resonance R_1ρ_ relaxation dispersion profiles of adenines C1′ atoms. R_1ρ_ (= R_2_ + R_ex_) rates of the C1′ atoms of adenines were plotted as a function of the effective spin-lock field power (ω1/2π). The experiments were performed at 25 °C. The two-state model fits (solid lines) were obtained using the protocol described in “Materials and Methods”. (R_2_ + R_ex_) standard deviations were calculated from the 500 runs carried out for each fit. The averaged R_ex_ values are specified in each panel.

A third series of experiments was carried out on the anomeric C1′ atoms. The (R_2_ + R_ex_) profiles (Fig. 5) and the R_2_ and R_ex_ values (Fig. 5 and Supplementary Table S2-3) show that the data related to the C1′ atoms of A_7_, A_8_, A_9_ and A_19_ are compatible with a slow conformational equilibrium. Among these four adenines, A_7_ and A_19_ are associated with especially high R_ex_ values (> 15 Hz) that exceed available measurements^15,17^ and reflect a relative abundance of excited state^3^. The flat R_1ρ_ profiles obtained for the C1′ atoms of other nucleotides, comprising thymine partners of adenines, correspond to much lower or null R_ex_ values (Supplementary Table S2-3).

When C1′ and C6–C8 relaxation dispersion experiments are available for the same nucleotide, R_2_ and R_ex_ values are consistent (Supplementary Table S2-2 vs Supplementary Table S2-3). Assuming that this property is true for all the nucleotides, the present relaxation dispersion experiments help to interpret the data subject to C8 atom resonance overlaps which concern in particular the nucleotide couples A_7_/A_19_ and A_9_/A_20_ (Fig. 4). Thus, both A_7_ and A_19_ likely contribute to C8 relaxation dispersion; the R_ex_ values, clearly higher for C1′ of A_9_ than for C1′ of A_20_, advocate for a major contribution of A_9_ to C8-R_ex_.

#### In sum

Null R_ex_ values show that there is no slow exchange on C·G base pairs, which is the norm at our pH conditions (pH 6.5)^42^. Also, the R_ex_ values calculated for the thymines paired with the five adenines do not furnish any robust evidence for motions (Supplementary Table S2). In contrast, non-null R_ex_ values appear on the five dodecamer adenines (Table 1). It is clear that A_7_ and A_19_ in T_6_pA_7_·T_18_pA_19_ are submitted to a slow conformational equilibrium, according to consistent, high R_ex_ values from C2, C8 and C1′ on-resonance experiments and to H2 and C2 linewidth broadenings. Among the three neighbouring adenines, A_8_ and A_9_ exhibit signs of dynamic events although the behaviour of their H2 and C2 atoms differ from those of A_7_ and A_19_; the case of A_20_ is much more disputable, given the low R_ex_ value associated with its C1′ atom.

**Table 1:** Adenines in T_5_T_6_A_7_A_8_A_9_·T_16_T_17_T_18_A_19_A_20_ associated with non-null R_ex_ value according to on-resonance relaxation dispersion experiments.

Atom type	High R_ex_ value	Low R_ex_ value
C2	R_ex_ ≥ 2.5 s^−1^: A_7_ A_19_	–
C8	R_ex_ ≥ 6.7 s^−1^: (A_7_ and/or A_19_) A_8_ (A_9_ and/or A_20_)	–
C1’	R_ex_ ≥ 6.0 s^−1^: A_7_ A_8_ A_9_ A_19_	R_ex_ ≤ 2.5 s^−1^: A_20_

On-resonance experiments are commonly used to provide information about the presence or absence of slow motion but by themselves they are insufficiently accurate for a viable quantification of the exchange parameters. Consequently, more sophisticated off-resonance experiments were also carried out to extract significant information about the conformational exchange process.

### Off-resonance R_1ρ_ dispersion relaxation experiments: characteristics of slow conformational exchange

Off-resonance dispersion relaxation experiments performed at a single magnetic field were used to obtain thermodynamic, kinetic and structural information about conformational exchange. This type of experiment was applied to the C1′ resonances of A_7_, A_8_, A_9_ and A_19_; the four adenines for which signs of exchange arise from on-resonance dispersion relaxation experiments. Additional investigations focused on T_6_, C_4_, G_15_ and G_21_, nucleotides associated to very low or null R_ex_ (Supplementary Table S2). C1′ resonances were chosen because they showed large signal to noise ratios and were subject to only a few overlaps so that data could be collected for most nucleotides of the dodecamer (Supplementary Tables S2, S3).

**Figure 6:**
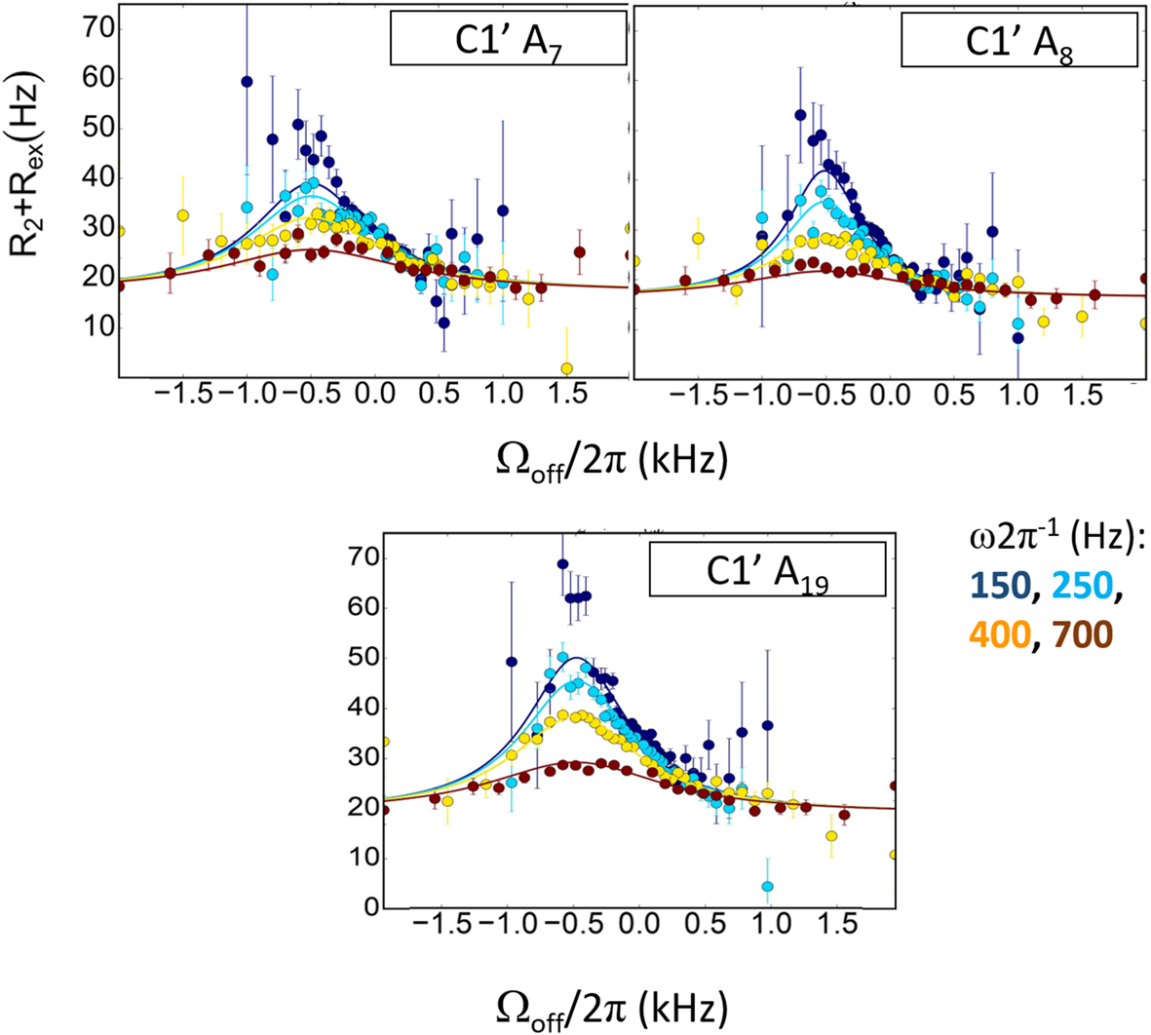
Off-resonance R_1ρ_ relaxation dispersion profiles for C1′ atoms of A_7_, A_8_ and A_19_. R_2_ + Rex (= R_1ρ_) values are given as a function of the resonance offset from the major state (Ω_off_/2π). Error bars represent experimental uncertainties. The experiments were carried out at four different spin-lock powers (From 150 to 700 Hz, colour code given in the bottom right of the Figure). The fits (solid lines) were performed using Method 2, described in “Materials and Methods”. The resulting exchange parameters are reported in Tables 2 and S3.

The off-resonance data are compatible with slow motions for A_7_, A_8_, and A_19_ alone (Fig. 6). A_9_, as well as the other nucleotides are associated with flat (R_2_ + R_ex_) profiles (examples in Supplementary Fig. S5). The A_7_, A_8_, and A_19_ (R_2_ + R_ex_) profiles were fitted using two distinct methods, an approach that enables to assess the robustness of the resulting parameters. We implemented first a classical analytic, algebraic method^15,43–45^, described as Method 1 in “Materials and Methods”, performing 1000 runs of calculations for each fit. The same data were also analysed using a completely different method recently published^3^, called here Method 2, which is based on the numerical integration of the Bloch-McConnell equations. Basically, both methods rely on a two-state exchange model and three variables, the rate exchange (k_ex_), the population of the minor conformer (p_E_) and the difference between the chemical shifts of major and minor conformers (Δω). Examples of fits with Method 2 are given in Fig. 6.

Applied to A_7_, A_8_ and A_19_ data, the exchange parameters from either Methods 1 or 2 are remarkably consistent (Tables 2 and S3), in the range of those previously published^15,17–20^. The rather modest standard deviations associated with the parameter values from Method 1 show the reliability of each run series. Nevertheless, some subtle differences are observed for the A_7_ exchange obtained from the two methods. Although it is clear that this nucleotide is submitted to a slow exchange, the A_7_-Δω value and standard deviation (4.16 ± 1.06 ppm) obtained from Method 1 appear too high compared to published data^15,17–20^. In addition, one expects a substantial p_E_ value, similar to that of A_19_, given their comparable high R_ex_ values that are primarily sensitive to the excited state population^3^. For these reasons, we prefer to give more weight to the A_7_-parameters from Method 2.

**Table 2:** A_7_, A_8_ and A_19_ conformational exchange parameters from C1′ off-resonance R_1ρ_ relaxation dispersion experiments.

C1′ nucleotide	k_ex_ (s^−1^)		p_E_ (%)		Δω (ppm)	
	Method 1	Method 2	Method 1	Method 2	Method 1	Method 2
A_7_	2991 ± 693	3309 ± 357	0.62 ± 0.19	0.80 ± 0.08	4.16 ± 1.06	3.32 ± 0.16
A_8_	1580 ± 306	1994 ± 199	0.58 ± 0.09	0.59 ± 0.02	3.53 ± 0.51	3.42 ± 0.1
A_19_	2680 ± 456	2872 ± 205	1.20 ± 0.01	1.00 ± 0.05	3.31 ± 0.32	3.29 ± 0.09

The exchange parameters presented in this Table correspond to the population of the minor conformer (p_E_), the rate exchange (k_ex_) and the difference between the chemical shifts of major and minor conformers (Δω); they were inferred from individual fits of C1′ off-resonance relaxation dispersion data according to a classical, home-made protocol (Method 1, see “Materials and Methods”) or an approach developed by Al Hashimi’s group (Method 2). Experimental data were collected at 600 MHz, 25 °C and pH 6.5.

The data related to A_7_ and A_19_ strengthen the interpretation of H2 and C2 linewidth broadening proposed above from on-resonance results. Indeed, there is now a clear parallel between H2 and C2 broadenings (Figs. 1 and 2), R_ex_ values (Fig. 3) and p_E_ (Table 2), all of these parameters being more accentuated for A_19_ than for A_7_. We therefore postulate that H2 and C2 broadenings not only reveal slow motions, but are also correlated with the excited state population. We will see below that this conclusion is very pertinent in the reconsideration of the results of previous 1D NMR experiments.

The case of A_9_ is intriguing and deserves a short comment. The R_ex_ profiles are either incompatible (off-resonance experiments, Supplementary Fig. S5) or compatible (on-resonance experiments, Fig. 5) with a conformational exchange. This unexpected situation has already been encountered^46^ and may correspond to a scenario in which the typical limits of slow exchange are no longer valid; in particular, A_9_ exchange rate could be faster than those of A_7_, A_8_ or A_19_.

The results above were obtained with NMR datasets from only C1′ sugar resonances. Because of numerous resonance overlaps, off-resonance data could only be collected for C8 and C2 atoms of A_19_, which allowed the constitution of a new extended dataset, involving sugar and base atoms. The fits of this dataset were performed assuming that C1′, C8 and C2 atoms are subjected to the same conformational exchange, with one unique value for k_ex_ and one unique value for p_E_. The resulting values of k_ex_, p_E_ and Δω are remarkably coherent with those obtained from C1′ resonances alone (Table 3), confirming in particular that the population of A_19_ minor conformer reaches 1.2/1.1%. From a methodological point of view, this approach shows that an off-resonance dataset from only one atom type is sufficient to produce reliable exchange parameters, with the obvious condition that the atom under consideration is implicated in the conformational transition of interest.

**Table 3:** A_19_ conformational exchange parameters from off-resonance R_1ρ_ relaxation dispersion experiments.

A_19_ atom(s)	k_ex_ (s^−1^)		p_E_ (%)		Δω (ppm) for C1’	
	Method 1	Method 2	Method 1	Method 2	Method 1	Method 2
C1′ alone	2680 ± 456	2872 ± 205	1.20 ± 0.01	1.00 ± 0.05	3.31 ± 0.32	3.29 ± 0.09
C1′, C8, C2 together	2671 ± 459	3138 ± 228	1.20 ± 0.20	1.10 ± 0.08	3.31 ± 0.31	3.31 ± 0.09

The exchange parameters presented in this Table correspond to the population of the minor conformer (p_E_), the rate exchange (k_ex_) and the difference between the chemical shifts of major and minor conformers (Δω); they were inferred from fits of off-resonance relaxation dispersion data according to Methods 1 or 2; the data are those collected from resonances of either C1′ alone or C1′, C8 and C2 together, at 600 MHz, 25 °C and pH 6.5.

The similarities between the two-state exchange characteristics of A_7_, A_8_ and A_19_ (Table 2) could be compatible with simultaneous transitions of the three nucleotides between ground and excited states, implying the transient co-existence of three successive base pair minor conformers. Thus, we completed our analyses by testing this hypothesis of synchronous motions. A collective transition implies that k_ex_ and p_E_ are identical for the three nucleotides and these conditions were therefore integrated as restraints in the fit calculations. This model provides reasonable values of conformational exchange parameters (Table 4) that, at first sight, are comparable to those obtained for individual motions (Table 2). Fisher tests were thus performed to evaluate which of the two hypotheses, non-simultaneous or simultaneous motions, led to the best result in terms of χ^*2*^. With both Methods 1 and 2, the F-values largely exceed the F-table value (Table 5). In accordance with the p-values that show the statistical significance of the tests, this means without ambiguity that the model assuming individual motions of each adenine is more effective at best representing the off-resonance data.

**Table 4:** Conformational exchange parameters from off-resonance R_1ρ_ relaxation dispersion experiments: model of coordinated motions of A_7_, A_8_ and A_19_.

	k_ex_ (s^−1^)		p_E_ (%)		Δω (ppm)	
	Method 1	Method 2	Method 1	Method 2	Method 1	Method 2
C1′ of A_7_, A_8_ and A_19_	2391 ± 299	2678 ± 153	0.85 ± 0.53	0.85 ± 0.11	3.32 ± 0.43	2.83 ± 0.11
					3.09 ± 0.33	2.59 ± 0.07
					3.71 ± 0.26	3.70 ± 0.09

The exchange parameters presented in this Table were produced by fitting a model in which the three nucleotides undergo coordinated motions; this hypothesis implies identical k_ex_ and p_E_ for the three nucleotides.

**Table 5:** Comparison between fits assuming coordinated or uncoordinated motion models for A_7_, A_8_ and A_19_.

	Degree of freedom	F-table value	F-value	p-value
Method 1	417	2.39	126	1.7 10^–70^
Method 2			48	2 10^–33^

Statistical F-test using χ^2^ and F-distribution analysis was performed to compare the fits based on models where A_7_, A_8_ and A_19_ undergo either coordinated, collective or uncoordinated, individual transitions; p-values validate the results.

Thus, the interpretation of the off-resonance dispersion relaxation experiments draws a picture of a block of three specific adenines that undergo slow motions between two states, the excited one representing from 0.6 to 1.2% of the conformers. While the three adenines are clustered in the T_6_A_7_A_8_·T_17_T_18_A_19_ segment, they adopt a non-synchronous regime of motions.

### Nature of the excited conformer

As mentioned above, both on- and off-resonance dispersion relaxation experiments and the inferred exchange parameters agree remarkably well with those obtained by Al Hashimi’s group within B-DNA sequences^15,17–20^. The asymmetry of the NMR data in A·T base pairs, in which evidence of slow dynamics is observable on adenines but not on their thymine partners, is also in line with these earlier results. It is now accepted that these NMR-based characteristics correspond to a dynamic equilibrium of particular base pairs between the canonical Watson–Crick (WC) conformation and an excited, short-lived state of low abundance, which is the Hoogsteen (HG) pairing^3,15,18,19^. HG base-pairing corresponds to N7_purine_ − N3_pyrimidine_ and N6/O6_purine_ − O4/N4_pyrimidine_ hydrogen bonds, which imply purine and pyrimidine nucleotides in syn- and anti-configurations, respectively. Thus, the WC → HG transition does not dramatically affect the pyrimidine but requires one major change, *i.e.* the slow motion of the purine around the glycosidic angle χ. Here, we can confidently attribute a WC ↔ HG equilibrium to the three successive A·T base pairs composing the T_6_A_7_A_8_·T_17_T_18_A_19_ segment. It should be underlined that the HG percentages calculated here for A_7_, A_8_ and A_19_ are among the highest measured populations^15,17–20^.

In addition to the purine rotation, the WC → HG transition is accompanied by some changes in sugar pucker and backbone torsion angles, which likely optimize hydrogen-bonding and stacking with neighbours^19,27,42^. In particular, a study of either WC or HG A·T containing oligomers showed that ^31^P signals are up-field shifted in and around HG A·T base pairs compared to their WC counterparts^42^. Such shifts are usually interpreted as being due to the presence of more BI conformers^47,48^.

Examining high-resolution X-ray structures of free DNA suggests that another backbone alteration could also occur on α/γ angles, as briefly previously mentioned^27^. Indeed, structures containing exclusively HG pairings (PDB codes 1GQU and 1RSB, of 2.5 and 2.2 Å of resolution, respectively) enclose TpA junctions adopting unusual α/γ:g+ /g− angles, instead of the canonical g-/g+ configuration. Unusual α/γ angles are infrequent in WC B-DNA X-ray structures^49,50^ because they are energetically very costly to generate^50^. Nevertheless, as rare as they are, these atypical α/γ conformations are mainly encountered in ApA steps or A·T-rich contexts (Supplementary Table S4). So, the potential ability of A·T-rich patches to adopt unusual α/γ conformations could be a factor promoting the emergence of HG base pairs.

### Sequence effect on TpA steps

The results presented above demonstrate that adenine H2 resonances associated with linewidth broadenings reflect the existence of a slow conformational exchange, as previously postulated^7,9,10^. However, any adenine submitted to slow motions is not associated with such anomalies that exclusively arise on TpA adenines. Accordingly, H2 linewidth broadenings are observed in the dodecamer on only those adenines belonging to TpA, A_7_ and A_19_ (Fig. 1). This specific phenomenon may be related to the especially high-field shifted H2 resonance of the major conformer (Fig. 1) that likely maximizes Δω, and consequently R_ex_—recalling that a high R_ex_ broadens the resonance.

This intimate connection between H2 linewidth broadening and slow motion allows the re-examination of earlier NMR data. A study reported measurements of H2 linewidth broadenings on 14 oligomers containing the 16 possible immediate sequence contexts of TpA steps (*i.e.* NTAN)^7,8^. This systematic approach showed that broadening of H2 occurs in diverse TpA environments, the maximal values corresponding to the central adenines of (Y/R)TAA tetramer fragments (Table 6).

**Table 6:** TpA steps associated with large H2 linewidth broadenings in the literature.

References	Oligomer sequence	A in TpA with max H2 linewidth > 15 Hz
McAteer et al. 1995	CTTT***A***^***1***^AATTT***A***^***2***^AAG	TT***A***^***1***^A (~ 15 Hz) and TT***A***^***2***^A (~ 19 Hz)
	CTTTACATGT***A***AAG	GT***A***A
	CTTTAGATCT***A***AAG	CT***A***A
	CTTTATATAT***A***AAG	AT***A***A
McAteer et al. 2000	GCTTATAT*A*AGC	AT***A***A
	GCATACGT***A***TGC	GT***A***T
	GCTTAGCT***A***AGC	CT***A***A
	GCTT***A***^***1***^ATT***A***^***2***^AGC	TT***A***^***1***^A (~ 18 Hz) and TT***A***^***2***^A (~ 22 Hz)
	GCTTACGT***A***AGC	GT***A***A

This table reports the sequences studied by Kennedy’s group in which the H2 linewidth broadening of one or two adenines (in bold, underlined) in TpA steps is equal or exceeds 15 Hz at 25 °C. The immediate environment of such TpA is reported in the last column. Note that all sequences are palindromic.

However, considering the nearest neighbour is insufficient to explain the slight but significant disparity of H2 linewidth broadenings of the TpA adenines that are in the same tetrameric environment in CTTTAAATTTAAAG^8^ and GCTTAATTAAGC^7^ (Table 6). Similarly, H2 broadening (Fig. 1) and slow motion characteristics differ between A_7_ and A_19_ in TTA_7_AA·TTA_19_A, for instance the population of the excited state (p_E_) (Table 2). These differences suggest a subtle, long-range influence of the sequence on the DNA’s ability to undergo WC ↔ HG transitions. At this stage, the only certitude is that slow DNA motions are modulated at dinucleotide and tetranucleotide levels, as previously established in solution for nano-second dynamics^37,47,51^.

## Discussion

This NMR study focused on a dodecamer containing the TTAAA·TTTAA fragment that was assumed to be an element facilitating histone anchoring upon nucleosome formation^34–37^. Slow conformational exchanges in the dodecamer were revealed and characterized from R_1ρ_ relaxation dispersion experiments. These NMR approaches also allowed a re-examination of classical 1D and 1D-T1 inversion-recovery experiments and to demonstrate the relationship between H2 linewidth broadenings and slow motions. Given that linewidth is easily observable and quantifiable, it represents an interesting means of detecting nucleotide slow dynamics, even if it appears to be limited to TpA adenine H2 protons.

As expected, most nucleotides of the dodecamer do not show consistent, conclusive signs of any slow motion. However, A_7_, A_8_, and A_19_ in the TA_7_A_8_·TTA_19_ fragment are specifically subjected to conformational exchange in the milli-second time range. The corresponding exchange parameters perfectly match the signature of WC ↔ HG base pair equilibrium as published by Al-Hashimi’s group^15,17–20^. Thus, we can confidently postulate that the TA_7_A_8_·TTA_19_ fragment is composed of three base pairs that transiently flip toward the HG conformation.

Although the crystallographic form of the ATTAAT·ATTAAT hexamer^52^ attests for the co-existence of three or more successive HG base pairs, our analyses agree with non-simultaneous WC ↔ HG transitions of the three A·T base pairs, which does not exclude a certain degree of cooperation between the motions. For instance, that one HG base pair locally destabilizes the double helix^42^ could favour a WC → HG flip of its closest neighbours. In any case, individual motions have the effect of magnifying the time during which one HG base pair is present in the TAA·TTA tract. Thus, according to the individual HG populations calculated here (~ 1% for each of the three HG base pairs), and given the non-simultaneous character of the motions highlighted by our fits, 3% of TAA·TTA fragment contains one HG A·T base pair.

This relatively high occurrence underlines the relevance of the question of an eventual function of transient HG base pairs in the nucleosome context, remembering that the TTAAA element likely helps histone anchoring. The first idea is that DNA wrapping could involve HG base pairs but our analyses failed to detect such pairing in high-resolution X-ray nucleosome structures, conversely to what was observed on several other types of DNA–protein complexes^27^. Another proposal relates to the exploitation of shape features specific to HG base pairs upon nucleosome formation. To our knowledge, there is only one structure ensemble based on NMR data that was collected on an oligomer containing one HG m^1^A·T base pair in the Am^1^ACC·GGTT environment^42^. This oligomer has as a main characteristic a major-groove kink. Indeed, analyses of X-ray structures of DNA bound to proteins or small molecules suggested that such major-groove curvature is induced by any HG base pair in any context^27,42^. This structural particularity cannot be considered as a favourable pre-organization for the nucleosome since TTAAA bound to the histone octamer presents a minor groove curvature^34,53^. In addition to the determination of the structure of our HG oligomer itself, another factor such as long-range consequence of HG base pair could be investigated. It has to be borne in mind that, at this point in time, there is no clear indication of how those HG base pairs present in the TAA element impact on nucleosome structure or dynamics. Indeed, it is now widely accepted that nucleosome assembly is primarily modulated by sequences that introduce structural variability for helical parameters such as roll or slide along the free WC B-DNA.

The percentage of HG conformers of the three A·T base pairs in T_6_A_7_A_8_·T_17_T_18_A_19_ is unusually high (~ 1%) for such minor, short-lived states that very rarely exceed 0.5%^15,17–20^. Multiple examples of enhanced dynamics on TA·TA adenines in the same TAA·TTA context emerge when one considers studies reporting linewidth broadenings (Table 4)^7,8^. However, the structural foundation for this specific, sequence dependent, stabilisation of HG base pairs remains unclear and poorly documented. One can only formulate hypotheses, being aware that such speculations will require future investigation. A first point concerns atypical features observed in A·T containing sequences. Thus, the photo reactivities of TT steps in TTAA·TTAA leading to T^T dimers are clearly outsized compared to those of any other dinucleotides, comprising other TT steps^53^; this particularity is added to (but cannot be totally explained by) the TTAA·TTAA marked positive rolls and low twists attested by both NMR^37,54^ and simulation^55^ data. Also, as highlighted in one of the above sections, A·T rich sequences show a specific propensity to adopt unusual α/γ backbone angle conformations (Supplementary Table S4). Such unusual structural elements could destabilize the WC form of A·T, favour the flipping-out of adenine from the double helix via the major groove^15,56^, which preludes WC ↔ HG conversions, and, in the case of α/γ angles, stabilize the HG base pair. Another suggestion emerges from the X-ray structure of the DNA bound to the MATα2 homeodomain^57^. In this complex, the T_6_A_7_A_8_·T_36_T_37_A_38_ fragment contains one HG base pair, A_7_·T_37_, in which A_7_ engages two hydrogen bonds with T_37_ and T_36_. A similar way to increase the stability of HG conformers may occur in any TAA·TTA fragment, in the present case via hydrogen bonds between A_7_A_8_ and T_16_T_17_T_18_ on the one hand and between A_19_ and T_5_T_6_ on the other.

Indeed, the effect of sequence in WC ↔ HG transition whilst incontestable remains complex and thus so far is only partially elucidated as already noted^17^. Further investigations are clearly required to clarify in particular, the effect of the tetra or even hexanucleotide contexts on the HG population. This topic is in fact essential for capturing the multiple aspects of DNA double helix functional versatility. A lot of information about nano-second dynamics keeping WC pairing intact is already available; these are primarily sensitive to the dinucleotide sequence while modulated at the tetramer level, as shown by experimental X-ray^49,58,59^ or NMR data^47,48,51,54^ and modelling^38,55,60^. NMR data in particular revealed that such rapid motions are especially enhanced in G·C rich elements^47,51,54^. Several examples demonstrated their importance in the structural adjustment of DNA to its protein partners, transcription factors^61–63^ or other proteins^37,51,64^. Beyond what occurs on WC G·C rich sequences at short timescales, A·T rich elements could be more specialized in milli-second conformational transitions, with the possibility of generating regions of Hoogsteen base-pair hot-spots that could play a topological role in genomic DNA^27^.

## Materials and methods

### Samples and resonance assignments

Samples were purchased as single-stranded oligonucleotides 5′-CCGCTTAAACGC-3′ and 5′-GCGTTTAAGCGG-3′ from Eurogentec (Belgium) for unlabelled DNAs or from Eurisotop (France) for fully ^15^N/^13^C–labeled DNAs. The two complementary strands were resuspended in 66 mM sodium phosphate buffer with 0.1 mM EDTA, for a total ionic strength of 0.1 M at pH 6.5; they were then mixed with a 1:1 ratio in 450 μL H_2_O. In a next step, the samples were lyophilized three times in 99.99% ^2^H_2_O. The final concentration of the unlabelled and labelled duplexes were 1.2 mM and 0.85 mM respectively in volumes of 500 μL and 180 μL respectively.

The full assignment of ^1^H and ^13^C resonances of the dodecamer were previously described^37,54^. To complete the carbon assignments and notably to obtain those of the quaternary carbons of bases, we performed additional experiments on a Bruker Avance spectrometer equipped with a 5-mm triple-resonance cryogenic probe, at 600 MHz frequency or 150 MHz for ^13^C experiments: (i) constant time ^1^H-^13^C HSQC optimized separately for aromatic and aliphatic carbons, C2 of adenine, C8 of purines, C5 and C6 of pyrimidines and C1′ of any base and, (ii) for the quaternary carbons C4, C5, C6 of adenines, 3D TROSY relayed HCCH-COSY^65^ according to Hansen et al.^14^.

### R_1ρ_ relaxation dispersion experiments

All experiments were performed on the same spectrometer as used for assignments, at pH 6.5 and 25 °C. As specified in the Results section, the linewidth broadening of H2-proton resonances is maximal at this temperature. 1D selective ^13^C R_1ρ_ pulse was applied as previously described^14,16^. The spin-lock powers (ω) needed to be carefully controlled, and were calibrated accordingly^14^. On-and off-resonance experiments were performed on C2, C6/C8 and C1′ atoms of the ^13^C/^15^N-labeled DNA. On-resonance data were recorded at various ω, from ~ 100 to 3,500 Hz; off-resonance data were collected at various spin-lock offset frequencies (Ω) and at three or four different spin-lock powers (ω). Details of these experiments are given in Supplementary Table S1. Ten delays were used to determine T_relax_, the monoexponential decrease of the magnetization: 0, 4, 8, 12, 12, 16, 20, 24, 32, 32 ms for C6, C8 and C2 carbons and 0, 4, 8, 12, 12, 18, 26, 34, 42 and 42 ms for C1′ carbons.

Note that the duplication of two delays in each experiment allowed an evaluation of errors in the measurement of the peak heights. These errors were subsequently used in Monte Carlo simulations to determine R_1ρ_ uncertainties. The data corresponding to Hartmann-Hahn matching were omitted from the fits of on-and off-resonance measurements as previously described^14^. The 1D dispersion data were processed using NMRPipe^66^.

### Equilibrium parameters from R_1ρ_ relaxation dispersion experiments

Our approach assumed a two–state equilibrium between a ground state G and a minor, excited state E in which p_G_ and p_E_, the G and E populations, are strongly asymmetric: pG ≫ pE. The equilibrium parameters that can be extracted from relaxation dispersion experiments are pG, pE, k_ex_ the exchange rate, Δω_GE_ the difference between the frequencies of G and E, and R_ex_, the quantity added to the relaxation rate due to exchange, which depends on p_G_, p_E_, k_ex_ and Δω_GE_.

#### On-resonance experiments

R_ex_ was obtained by fitting the profiles of data from on-resonance experiments carried out in function of spin-lock offset powers (ω). In these experiments, θ, the angle between the effective field and the z axis, has the particular value of π/2. The fit is based on Eq. (1), a version of Eq. (2) that is simplified by using θ = π/2 to represent the condition of on-resonance experiments. For each fit, 500 runs were performed using R_1ρ_ values.

1 $$R_{1\rho } = R_{2} + R_{ex} = R_{2} + \frac{{\Phi_{ex} k_{ex} }}{{\omega_{1}^{2} + k_{ex}^{2} }};\;\;\;\Phi_{ex} = p_{G} p_{E} \Delta \omega_{GE}^{2}$$

R_1_ and R_2_ are the intrinsic longitudinal and transverse relaxation rates, respectively, which are supposed to be identical for the two-states G and E; ω_1_ is the spin-lock power.

#### Off-resonance experiments

The profiles of the off-resonance relaxation dispersion data measured in function of spin-lock offset frequencies (Ω) and power (ω) were fitted by two methods described below.

Method 1 is based on the Eq. (2)^15,43–45^.

2 $$R_{1\rho } = R_{1} \cos^{2} \theta + R_{2} \sin^{2} \theta + \sin^{2} \theta \frac{{p_{G} p_{E} \Delta \omega_{GE}^{2} k_{ex} }}{{\Omega_{E}^{2} + \omega_{1}^{2} + k_{ex}^{2} }}$$

In this case, restraints were applied on R_2_: R_2_ ≥ 16 s^−1^ and R_2_ ≤ 19 s^−1^, according to the range inferred from on-resonance experiments; Ω_E_ and Ω_G_ are the resonance offsets for the excited and the ground states E and G, respectively; Δω_GE_ = Ω_E_ − Ω_G_;

ω_1_ is the spin-lock power related to by the relation

$${\tan}\theta = \omega_{{1}} /\Omega_{{{\text{ave}}}} ,\;{\text{with}}\;\Omega_{{{\text{ave}}}} = \Omega_{{\text{E}}} {\text{p}}_{{\text{E}}} + \Omega_{{\text{G}}} {\text{p}}_{{\text{G}}} \,{\text{and}}\;{\text{ p}}_{{\text{E}}} + {\text{p}}_{{\text{G}}} = { 1}.$$

k_ex_ = k_G_ + k_E_ is the exchange rate with k_G_ = p_E_k_ex_ and k_E_ = p_G_k_ex_, k_G_ and k_E_ representing the forward and reverse rate constants, respectively.

For each fit, 1000 runs were performed using R_1ρ_ values. The R_1ρ_ values from off-resonance experiments were systematically and randomly varied using standard deviations derived from the fits of the exponentially decreasing intensities of the peaks as function of the relaxation time. This protocol implemented using home-based Python and R scripts enables to derive the errors associated to each exchange parameter according to a strategy described previously^67^.

Method 2 used the “Bloch-McConnell Numerical Simulator” (BMNS) developed by Al-Hashimi’s group^3^, which is exclusively based on a two-state exchange model. The calculations were performed with the same constraints on the R_2_ relaxation rates than for the fits described above.

## Supplementary information

Supplementary Information.
