# Supplementary material for: Slow motions in A·T rich DNA sequence
Source: Sci Rep. 2020 Nov 4;10:19005. doi: 10.1038/s41598-020-75645-x (PMC7642443; doi:10.1038/s41598-020-75645-x)
Supplement: Supplementary file 1 — Supplementary Information. [file 41598_2020_75645_MOESM1_ESM.pdf]

Slow motions in A•T rich DNA sequence

Ben Imeddourene[1], A., Zargarian[1], L., Buckle[1], M., Hartmann[1], B., & Mauffret[1,*], O.

Figures:

Figure S1: 1D and 1D-T1 inversion recovery spectra of the DNA aromatic region.

Figure S2. Chemical shift variations of H2 adenine protons as a function of temperature.

Figure S3: Examples of mono-exponential decays of relative peak intensities.

Figure S4: Examples of on-resonance ^{13}C6/8 $R_{1\rho}$ relaxation dispersion profiles for nucleotides not submitted to conformational exchange.

Figure S5**:** Examples of off-resonance ^{13}C1' $R_{1\rho}$ relaxation dispersion profiles for nucleotides not submitted to conformational exchange of A_9 and G_{21}.

Tables:

Table S1: Parameters used for the R1ρ relaxation dispersion experiments.

Table S2: R_2 and R_{ex} values from fits of on-resonance $R_{1\rho}$ relaxation dispersion experiments.

Table S3: Conformational exchange parameters from off-resonance $R_{1\rho}$ relaxation dispersion experiments.

Table S4: Unusual α/γ backbone angles in X-ray structures of decamers.

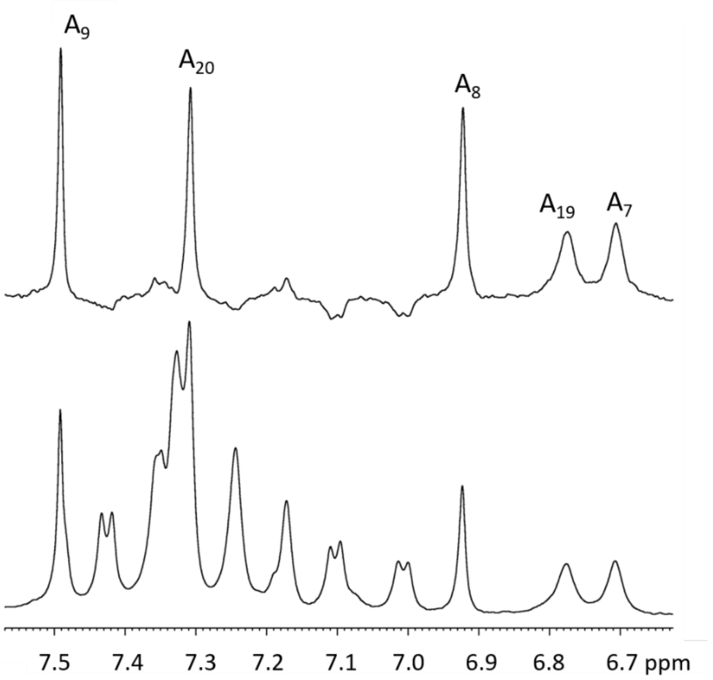

Figure S1: 1D and 1D-T1 inversion recovery spectra of the DNA aromatic region.

A: 1D (bottom) and 1D-T1 inversion recovery (top) spectra of the aromatic region of the unlabeled oligomer at 25°C in $^{2}H_2O$. The inversion recovery delay was optimized to selectively visualize the H2 resonances of adenines, which showed large differences in T1 values. The adenine numbering is recall on the top of the Figure.

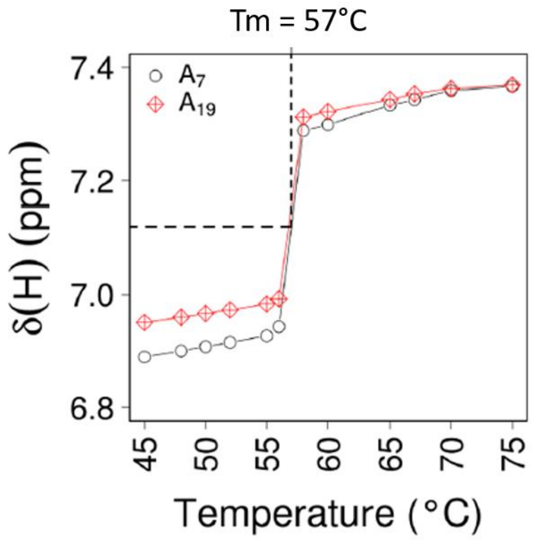

Figure S2. Chemical shift variations of H2 adenine protons as a function of temperature.

The variations of the H2 chemical shifts (δH) of A_7 and A_{19} located at the dodecamer center, were plotted as a function of temperature; accordingly, the Tm of the studied dodecamer is 57°C.

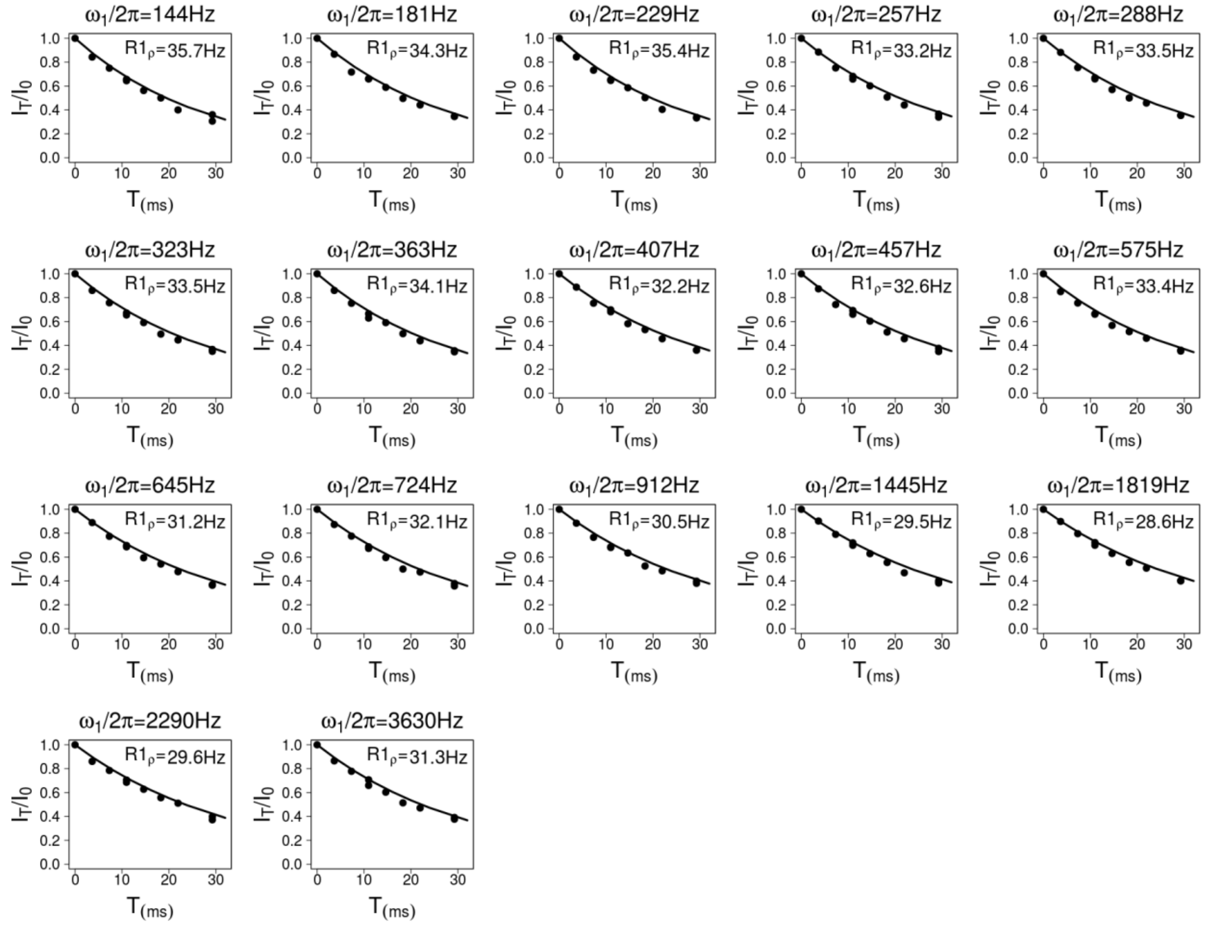

Figure S3: Examples of mono-exponential decays of relative peak intensities.

The mono-exponential decays of relative peak intensities presented here were measured on the C2 atom of the adenine A_{19}. I_T is the peak intensity of the C2 atom of A_{19} at each measured relaxation time T in millisecond (ms); I_0 is the intensity at T=0. The I_T/I_0 ratio is plotted as a function of T, from experiments performed with various spinlock powers ($\omega_1/2\pi$) specified above each panel. The R1ρ values given within the panels were obtained by fitting the decay curves with the equation $I_T = I_0 \exp(-RT)$, R being the time constant of the exponential.

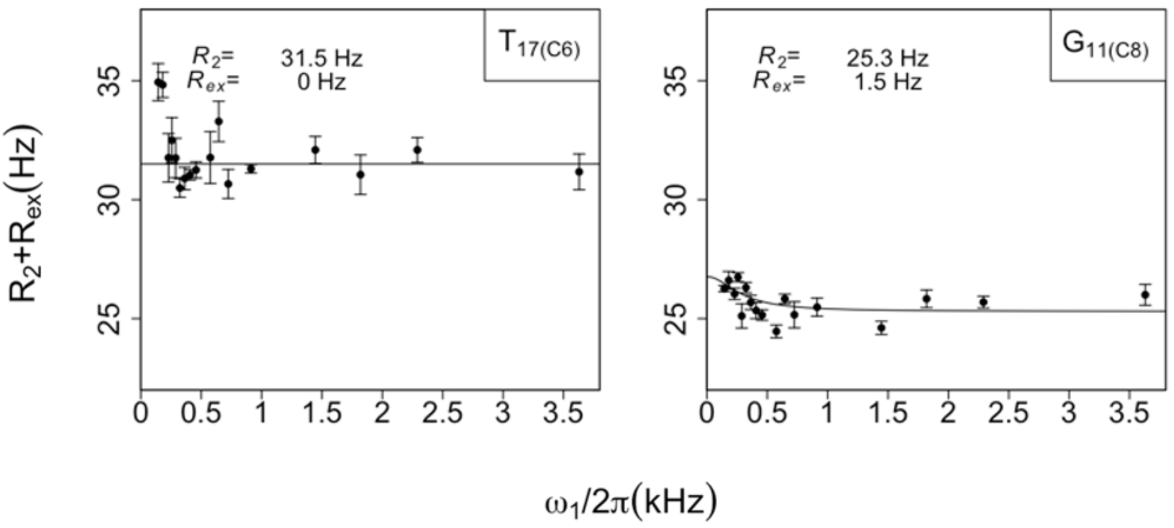

Figure S4: Examples of on-resonance ^{13}C6/8 $R_{1\rho}$ relaxation dispersion profiles for nucleotides not submitted to conformational exchange.

$R_{1\rho}$ (= R_2 + R_{ex}) rates of the T_{17}-C6 and G_{11}-C8 atoms were plotted as a function of the effective spin lock field strength (ω1/2Π). The experiments were performed twice at 25°C. The two-state model fits (solid lines) were obtained using the protocol described in Materials and Methods. (R_2 + R_{ex}) standard deviations were calculated from the 500 runs carried out for each fit. The averaged R_2 and R_{ex} values are specified in each panel.

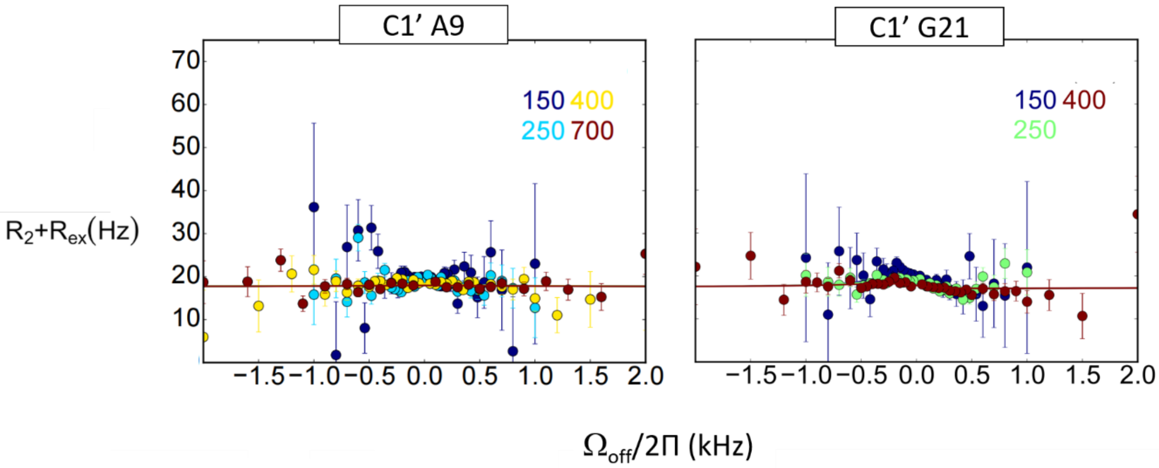

Figure S5: Off-resonance ^{13}C $R_{1\rho}$ relaxation dispersion profiles for C1’ atoms of A_9 and G_{21}.

R_2 + Rex (= $R_{1\rho}$) values are given as a function of the resonance offset from the major state ($\Omega_{off}/2\Pi$). Error bars represent experimental uncertainties. The experiments were carried out at four different spin lock powers (from 150 to 700 Hz, color code in insets).

Table S1 : Parameters used for the R1ρ relaxation dispersion experiments.

On resonance experiments

1- ***Aromatic carbon probes (N: nucleotide, Cx: aromatic carbon) :***

C_1 C6, C_2 C6, G_3/G_{15} C8, C_4 C6, T_5 C6, T_6/T_{18} C6, A_7/A_{19} C8, A_7 C2, A_{19} C2, A_8 C8, A_8 C2, A_9/A_{20} C8, A_9 C2, A_{20} C2, C_{10} C6, G_{11} C8, C_{12} C8, G_{13} C8, C_{14} C6, G_{15}/G_3 C8, T_{16} C6, T_{17} C6, G_{21} C8, C_{22} C6, G_{23} C8; the slash symbol indicates overlapping signals in the HSQC spectra.

17 values of spin lock power (ω): 144, 181, 229, 257, 288, 323, 363, 407, 457, 575, 645, 724, 912, 1445, 1819, 2290, 3630 Hz

2- ***C1' probes :***

C_1 C1', C_2 C1', G_3/G11 C1', C_4 C1', T_5 C1', T_6 C1', A_7 C1', A_8 C1', A_9 C1', C_{10} C1', C_{12} C1', G_{13} C1', C_{14} C1', G_{15} C1', T_{16} C1', T_{17} C1', T_{18} C1', A_{19} C1', A_{20} C1', G_{21} C1', C_{22} C1', G_{23} C1', G_{24} C1'; the slash symbol indicates overlapping signals in the HSQC spectra.

15 values of spin lock power (ω): 229, 257, 288, 323, 363, 407, 457, 575, 645, 724, 912, 1445, 1819, 2290, 3630 Hz

Off resonance experiments

Off resonance spin lock power (ω) & ± {offset (Ω), 36 values}

1- ***Adenine C1' probes :*** A_7 C1', A_8 C1', A_9 C1', A_{19} C1', A_{20} C1'

150 Hz & ±{30,60,90,120,150,180,210,240,270,300,360,420,480,540,600,700,800,1000}

250 Hz & ±{30,60,90,120,150,180,210,240,270,300,360,420,480,540,600,700,800,1000}

400 Hz & ±{50,100,150,200,250,300,350,400,450,500,600,700,800,900,1000,1200,1500,2000}

700 Hz & ±{100,200,300,400,500,600,700,900,1100,1300,1600,2000,2500,3000,3500,4000,5000,7500}

2- ***Other C1' probes :*** C_4 C1', G_{15} C1', G_{21} C1'

150 Hz & ±{30,60,90,120,150,180,210,240,270,300,360,420,480,540,600,700,800,1000}

250 Hz & ±{30,60,90,120,150,180,210,240,270,300,360,420,480,540,600,700,800,1000}

400 Hz & ±{50,100,150,200,250,300,350,400,450,500,600,700,800,900,1000,1200,1500,2000}

Table S2 : R_2 and R_{ex} values from fits of on-resonance $R_{1\rho}$ relaxation dispersion experiments

The R_2 and R_{ex} values reported in these Tables were obtained from fits of on-resonance ^{13}C $R_{1\rho}$ relaxation dispersion data collected for three carbon types, C2, C6 or C8, and C1'. Standard deviations were estimated from duplicate data. Two nucleotides separated by a slash (N/N) indicate overlapping data. The data related to the five adenines of the dodecamer are stressed by a grey background.

Table S2-1

atom	nucleotide	R_2 (s^{-1})	R_{ex} (s^{-1})
C2	A_7	30.2 ± 0.4	3.0 ± 1.4
	A_8	30.3 ± 0.3	0.8 ± 0.4
	A_9	30.2 ± 0.1	0
	A_{19}	28.2 ± 0.7	6.2 ± 1.8
	A_{20}	30.5 ± 0.2	0

Table S2-2

atom	nucleotide	R_2 (s^{-1})	R_{ex} (s^{-1})
C6	C_1	28.7 ± 0.2	0
C6	C_2	31.8 ± 0.4	2.0 ± 1.2
C6	C_4	33.3 ± 0.1	0
C6	T_5	30.5 ± 0.5	0
C8	A_8	26.7 ± 0.2	7.4 ± 0.5
C6	C_{10}	33.5 ± 0.7	0
C8	G_{11}	25.3 ± 0.2	1.5 ± 0.5
C6	C_{12}	32.7 ± 0.2	0
C6	C_{14}	33.6 ± 0.2	2.3 ± 0.6
C6	T_{16}	31.8 ± 0.8	0
C6	T_{17}	31.5 ± 0.6	0
C6	C_{22}	32.9 ± 0.3	2.0 ± 0.8
C8	G_{24}	27.0 ± 0.1	0
C8	G_3/G_{15}	27.9 ± 0.5	0
C6	T_6/T_{18}	31.6 ± 0.2	3.9 ± 0.7
C8	A_7/A_{19}	26.9 ± 0.2	8.4 ± 0.7
C8	A_9/A_{20}	26.6 ± 0.2	6.7 ± 0.5

Table S2-3

atom	nucleotide	R_2 (s^{-1})	R_{ex} (s^{-1})
C1’	C_1	10.5 ± 0.2	0
	C_2	16.2 ± 0.3	0
	C_4	17.4 ± 0.2	0
	T_5	17.3 ± 0.2	4.9 ± 0.4
	T_6	15.9 ± 0.1	2.7 ± 0.5
	A_7	17.3 ± 0.4	15.1 ± 0.7
	A_8	17.5 ± 0.3	7.5 ± 0.8
	A_9	18.0 ± 0.1	6.0 ± 0.2
	C_{10}	16.7 ± 0.2	3.6 ± 0.4
	C_{12}	16.3 ± 0.2	0
	G_{13}	12.2 ± 0.3	0
	C_{14}	15.5 ± 0.2	0
	G_{15}	18.5 ± 0.2	0
	T_{16}	17.6 ± 0.3	3.5 ± 0.8
	T_{17}	19.7 ± 0.2	0
	T_{18}	18.6 ± 0.3	0
	A_{19}	18.7 ± 0.2	15.9 ± 0.2
	A_{20}	17.2 ± 0.1	2.5 ± 0.2
	G_{21}	17.7 ± 0.1	0
	C_{22}	17.2 ± 0.2	0
	G_{23}	16.1 ± 0.1	0
	G_{24}	14.4 ± 0.1	0

Table S3: A_7, A_8 and A_{19} conformational exchange parameters from off-resonance $R_{1\rho}$ relaxation dispersion experiments.

The exchange parameters presented here complete those of the Table 2. R_1 and R_2 are the intrinsic longitudinal and transverse relaxation rates, respectively. These data were extracted using individual fits of experimental off-resonance relaxation dispersion data collected at 600 MHz, 25°C and pH 6.5.

		R_1 (s^{-1})		R_2 (s^{-1})	
		Method 1	Method 2	Method 1	Method 2
**C1'**	A_7	1.51 ± 0.29	1.44± 0.09	17.74 ± 1.39	17.13 ± 0.99
	A_8	1.43 ± 0.31	1.26 ± 0.10	16.86 ± 1.24	16.36 ± 0.42
	A_{19}	1.92 ± 0.18	1.76 ± 0.07	16.94 ± 1.31	19.0 ± 0.63

Table S4: Unusual α/γ backbone angles in X-ray B-DNA structures

The examined dataset is composed of 33 high resolution (R ≤ 2 Å) X-ray B-DNA structures, without mismatch, modified bases or phosphates, or non-paired bases. This Table reports the seven oligomers that contain unusual α/γ backbone angles (in bold and italic, underlined).

PDB code	sequence	<u>***α/γ***</u>
1IKK	CCTTTAAAGG	
	GGA<u>***AA***</u>TTTCC	<u>***g+/t***</u>
1SK5	CTTTT<u>***AA***</u>AAG	<u>***g+/t***</u>
	GAAAATTT<u>***TC***</u>	<u>***g+/t***</u>
1D49	CGATTAATCG	
	GCT<u>***AA***</u>TTAGC	<u>***g+/t***</u>
126D	CATGGCCATG	
	GTA<u>***CC***</u>GGTAC	<u>***g+/g-***</u>
307D	C<u>***AA***</u>AGA<u>***AAA***</u>G	<u>***g+/g- * 3***</u>
	GTTTCTTTTC	
307D	C<u>***AA***</u>AGA<u>***AAA***</u>G	<u>***g+/g- * 3***</u>
	GTTTCTTTTC	
1ZFG	CCGAG<u>***CT***</u>CGG	<u>***g+/g-***</u>
	GGC<u>***TC***</u>GAG<u>***CC***</u>	<u>***g+/g- * 2***</u>
